# Fatty Acid- and Cholesterol Transporter Protein Expression along the Human Intestinal Tract

**DOI:** 10.1371/journal.pone.0010380

**Published:** 2010-04-29

**Authors:** Christiaan J. Masson, Jogchum Plat, Ronald P. Mensink, Andrzej Namiot, Wojciech Kisielewski, Zbigniew Namiot, Joachim Füllekrug, Robert Ehehalt, Jan F. C. Glatz, Maurice M. A. L. Pelsers

**Affiliations:** 1 Department of Human Biology, NUTRIM School for Nutrition, Toxicology and Metabolism, Maastricht University Medical Centre+, Maastricht, The Netherlands; 2 Department of Human Anatomy, Medical University of Bialystok, Bialystok, Poland; 3 Department of Clinical Pathomorphology, Medical University of Bialystok, Bialystok, Poland; 4 Department of Physiology, Medical University of Bialystok, Bialystok, Poland; 5 Department of Gastroenterology, University Hospital Heidelberg, Heidelberg, Germany; 6 Department of Molecular Genetics, CARIM School for Cardiovascular Diseases, Maastricht University Medical Centre+, Maastricht, The Netherlands; 7 Department of Clinical Chemistry, Maastricht University Medical Centre+, Maastricht, The Netherlands; University of Cambridge, United Kingdom

## Abstract

**Background:**

Protein distribution profiles along the human intestinal tract of transporters involved in the absorption of cholesterol and long-chain fatty acids (LCFA) have been scarcely evaluated.

**Methodology/Principal Findings:**

In post-mortem samples from 11 subjects, intestinal transporter distribution profiles were determined via Western Blot. Differences in transporter protein levels were statistically tested using ANOVA and Tukey's Post Hoc comparisons. Levels in all segments were expressed relative to those in duodenum. Except for ABCG5 and FATP4, levels (mean±SEM) were the highest in the ileum. For ABCA1, ileal levels (1.80±0.26) differed significantly from those in duodenum (P = 0.049) and proximal colon (0.92±0.14; P = 0.029). ABCG8 levels in ileum (1.91±0.30) differed from those in duodenum (P = 0.041) and distal colon (0.84±0.22; P = 0.010) and jejunum (1.64±0.26) tended to be higher than distal colon (0.84±0.22; P = 0.087). Ileal NPC1L1 levels (2.56±0.51) differed from duodenum levels (P = 0.019) and from distal colon (1.09±0.22; P = 0.030). There was also a trend (P = 0.098) for higher jejunal (2.23±0.37) than duodenal NPC1L1 levels. The levels of ABCG5 did not correlate with those of ABCG8. FAT/CD36 levels in ileum (2.03±0.42) differed from those in duodenum (P = 0.017), and proximal and distal colon (0.89±0.13 and 0.97±0.15 respectively; P = 0.011 and P = 0.014). FABPpm levels in ileum (1.04±0.13) differed from proximal (0.64±0.07; P = 0.026) and distal colon (0.66±0.09; P = 0.037).

**Conclusions/Significance:**

The distribution profiles showed a bell-shape pattern along the GI-tract with the highest levels in ileum for ABCA1, ABCG8, NPC1L1, FATCD36 and FABPm, suggesting a prominent role for ileum in transporter-mediated uptake of cholesterol and LCFAs.

## Introduction

The incidence of the metabolic syndrome (MS) has rapidly increased over the last few decades [1], [2]. MS is a clustering of metabolic risk markers, including abdominal obesity, elevated plasma glucose levels, and an atherogenic lipid profile, which altogether contribute to the development of cardiovascular disease (CVD) [3], [4]. Patients suffering from the MS often show disturbances in fatty acid (FA) metabolism [5] leading to elevated plasma free fatty acid levels which negatively influence insulin-mediated glucose uptake [6], [7]. The disturbances in lipoprotein profiles most likely originate from an elevated hepatic production of large triacylglycerol-rich VLDL1 particles, which in combination with increased cholesterylester transfer protein (CETP) mediated lipid fluxes and decreased lipoprotein lipase (LPL) mediated lipolysis results in hypertriglyceridemia and low serum HDL cholesterol concentration [8], [9], [10]. However, it becomes more and more evident that lipoprotein metabolism is also regulated by absorption characteristics of cholesterol and FA in the intestine. For example, it was recently shown that the level of cholesterol absorption from the intestine was inversely related to reverse cholesterol transport from peripheral tissue macrophages into the feces [11].

Concerning the MS, there is an ongoing discussion whether these patients are characterized by elevated and/or accelerated intestinal cholesterol absorption or not. In this respect, Miettinen *et al*. [12] have proposed, that subjects can be characterized as cholesterol absorbers (with a low cholesterol synthesis) or as cholesterol synthesizers (with a low cholesterol absorption). Based on circulating levels of plant sterols which can be used as markers for fractional cholesterol absorption, it has been suggested that subjects with the MS are rather cholesterol synthesizers than absorbers [13]. Indeed, obese subjects displayed increased cholesterol synthesis and a decreased fractional cholesterol absorption [14].

**Figure 1:**
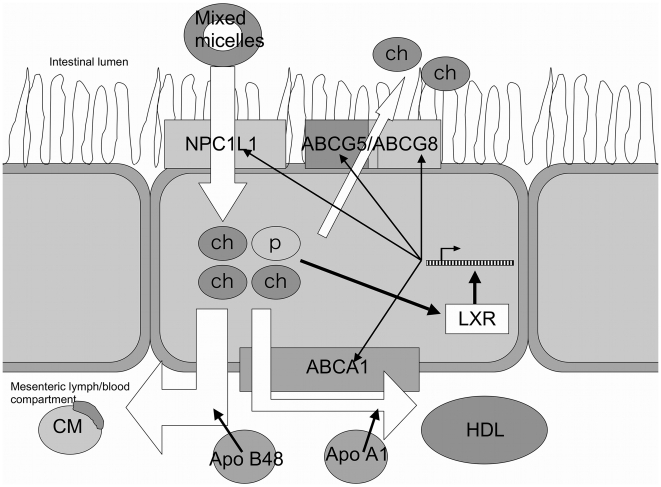
Intestinal uptake of dietary cholesterol. Dietary cholesterol (ch) and plant sterols/stanols (ps) travel, incorporated in mixed micelles, through the intestinal lumen. The sterols are transported across the brush-border membrane by NPC1L1. Once taken up, sterols are either incorporated in apoB48-rich chylomicrons (CM), which are secreted in the lymph compartment, or used to form apoA1-rich HDL cholesterol, a process that is mediated by ABCA1. Excess amounts of sterols are also excreted into the intestinal lumen by the reverse sterol transporters ABCG5 and G8. The level of transport proteins is under tight control of the liver X receptor (LXR) gene. This gene indirectly measures cellular sterol levels and regulates the transcription of sterol transporters NPC1L1, ABCA1, ABCG5 and G8.

Intestinal cholesterol absorption is tightly regulated by a number of transporter proteins and whether an individual is a cholesterol absorber or cholesterol synthesizer might be influenced by variations in the intestinal protein levels or activities of these cholesterol transporters. Four important proteins involved in transporting sterols across the intestinal lining are (1) adenosine-triphosphate (ATP) binding cassette A1 (ABCA1), a 226 kDa basolateral membrane protein which is involved in the synthesis of HDL by basolateral donation of cholesterol to its acceptor apolipoprotein A1; (2) ATP binding cassette G5 (ABCG5) and (3) G8 (ABCG8), also called sterolin-1 and sterolin-2 respectively, both with a molecular weight of ∼65 kDa. ABCG5 and ABCG8 are expressed apically at the brush border membrane [15] and collaborate in transporting excess sterols out of the enterocyte back into the lumen [16]. The fourth protein is Niemann-Pick C1 like 1 (NPC1L1), a 150 kDa protein which may cooperate with the scavenger receptor class B type 1 (SR-BI) [17] and regulates the intestinal uptake of sterols (figure 1). In addition, some recent studies suggest that several FA transporters, i.e. membrane fatty acid translocase (FAT/CD36) and cytoplasmic fatty acid-binding protein (I-FABP and L-FABP), are also involved in the uptake of cholesterol, making cholesterol and long-chain fatty acid (LCFA) uptake potentially interrelated [18], [19], [20].

**Figure 2:**
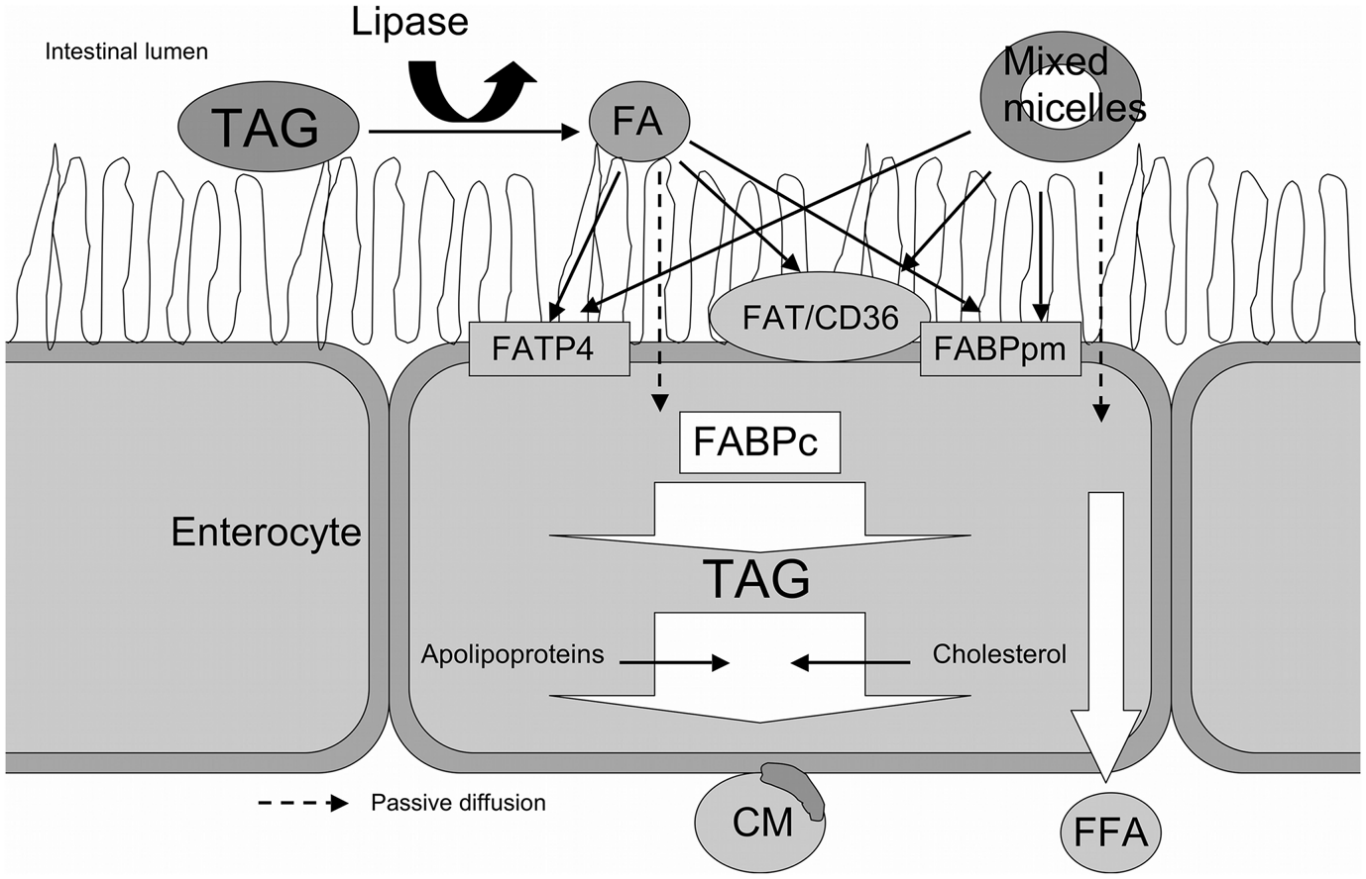
Intestinal uptake of dietary fatty acids. Dietary fatty acids (FA) pass through the intestinal lumen whilst esterified to triacylglycerols (TAG) or incorporated into mixed micelles. Gastric and hepatic lipases free the fatty acids, which are then receptive to uptake. Fatty acids are either transported across the apical membrane actively by FATP4, FAT/CD36 or FABPpm, or passively diffuse (blocked arrows) through the lipid bilayer. In the enterocyte FABP_c_ facilitates fatty acid transport through the cytosol. In the cytoplasm, the major part of fatty acids is re-esterified to triacylglycerols and excreted into chylomicrons (CM), whereas a small part is excreted as free fatty acids (FFA).

Important intestinal FA transporters are (1) FABPpm, a 40 kDa protein located peripherally on the plasma membrane and identical to the mitochondrial enzyme aspartate aminotransferase [21]; (2) FAT/CD36, an 88 kDa integral membrane glycoprotein with two predicted transmembrane domains, also known as the Class B scavenger receptor CD36 [22] and (3) fatty acid transport protein subtype 4 (FATP4), a 63 kDa integral membrane protein which possibly can drive fatty acid uptake or activate FA by trapping them inside the cell as their CoA thioesters [23], [24], [25], [26], [27]. Once LCFA are taken up, they can be transported to the mitochondria via cytoplasmic fatty acid-binding proteins (FABP_c_) for β-oxidation (figure 2) [28], [29]. These proteins also facilitate the cellular uptake of FA and protect against the cytotoxic effects of free cellular FA [30].

Data concerning differences in the intestinal levels of these transporter proteins between MS patients and healthy controls is not available. However, new strategies to improve serum lipoprotein profiles could focus on the role of the intestine in dyslipidemia [31], [32]. Therefore, it is important to know the intestinal protein distribution profiles of these transporter proteins in order to develop possible site-specific modulators of intestinal fatty acid and sterol metabolism. For these reasons, we decided to investigate the protein levels of the above-mentioned transporters which were chosen based upon recent up-to-date reviews, in different segments of the intestinal tract to visualize their distribution profiles along the human duodenal-colonal axis.

## Results

### (Chole)sterol transport proteins

**Figure 3:**
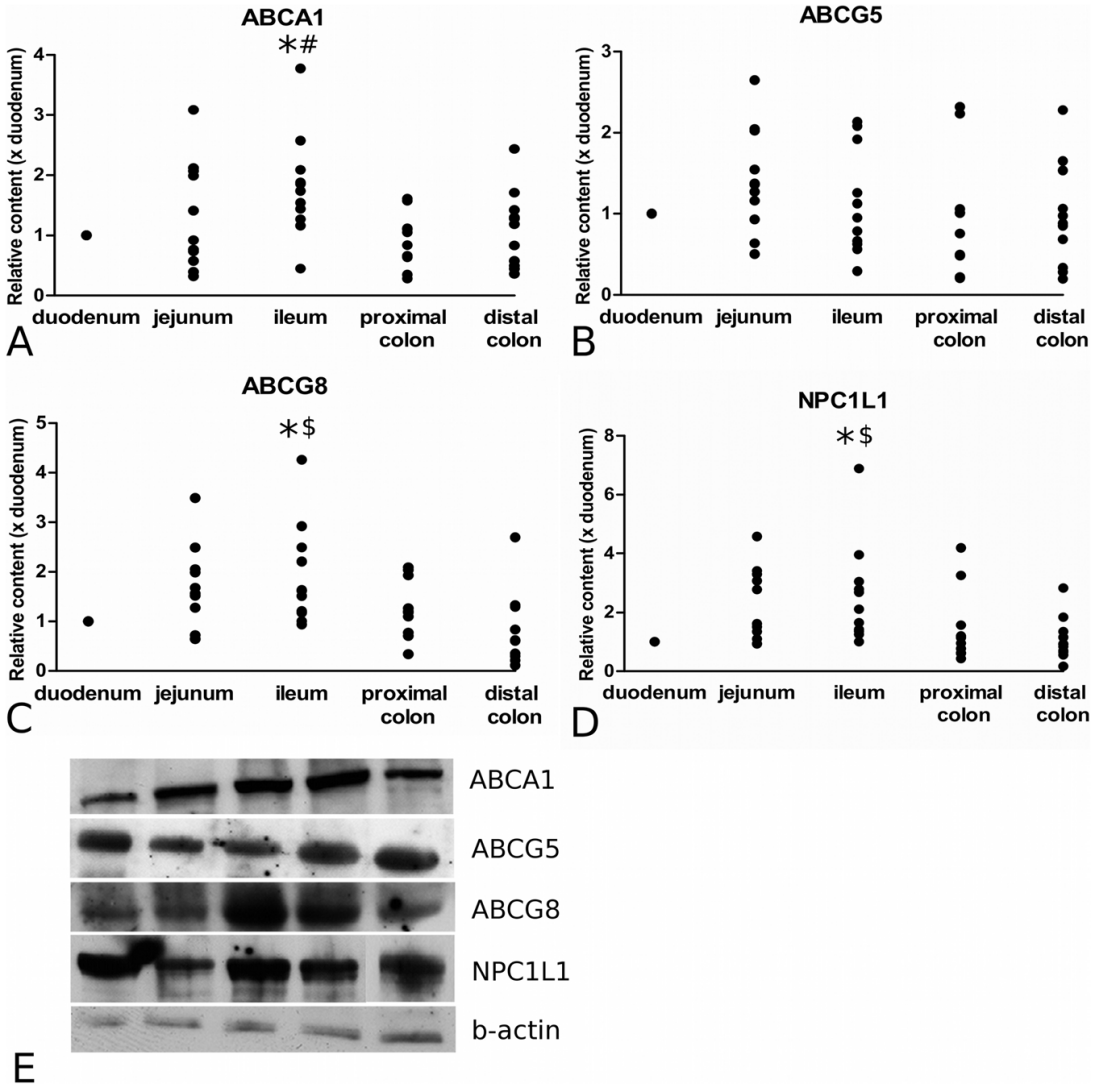
Distribution patterns of cholesterol transporter proteins along the longitudinal axis of the human intestine. These transporter proteins included ABCA1 (panel A), ABCG5 (panel B), ABCG8 (panel C) and NPC1L1 (panel D). Panel E displays Western Blots of the different segments of a single subject, which is representative for the average distribution pattern of ABCA1, ABCG5 and ABCG8 and NPC1L1. * = P<0.05 compared to duodenum; # = P<0.05 compared to proximal colon; $ = P<0.05 compared to distal colon. D = duodenum, J = jejunum, I = ileum, PC = proximal colon and DC = distal colon.

As explained in the method section, the level of each individual protein in duodenum was arbitrarily set at 1. ABCA1 protein level was significantly higher in ileum (1.80±0.26) than in duodenum (P = 0.049) and proximal colon (0.92±0.14; P = 0.029) (figure 3A). For ABCG5, no significant differences between duodenum, jejunum (1.41±0.19), ileum (1.13±0.20), proximal colon (0.98±0.24) and distal colon (0.98±0.19) were found (figure 3B). Ileal level of ABCG8 was significantly higher (1.91±0.30) than that in duodenum (P = 0.041) and distal colon (0.84±0.22; P = 0.010) and also tended to be higher in jejunum (1.64±0.26) than in distal colon (P = 0.087) (figure 3C). The protein level of the cholesterol transporter Niemann-Pick C1 Like 1 was significantly higher in ileum (2.56±0.51) as compared to that in duodenum (P = 0.019) and in distal colon (1.09±0.22; P = 0.030), whereas jejunum NPC1L1 level (2.23±0.37) tended to be higher than distal colon (P = 0.098). (figure 3D). Blots of individual subjects representative for the group are presented in figure 3E. Protein levels of the various sterol transporters did not differ between men and women, and did not correlate with age. There was no significant correlation between ABCG5 and ABCG8.

Concerning possible influence of protein degradation in the samples, linear regression analysis between time of biopsy after death and expression levels of ABCA1, ABCG8, NPC1L1 and FAT/CD36 did not show any association, whereas even a positive relation was found for ABCG5 (R^2^ = 0.25; P = 0.0001). Additionally, this analysis was performed per intestinal segment, but the results were comparable (data not shown).

### Long-chain fatty acid transport proteins

**Figure 4:**
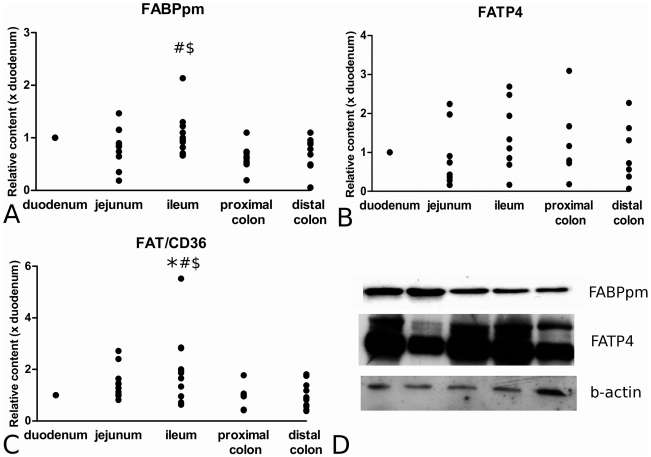
Distribution patterns of fatty acid transporter proteins along the longitudinal axis of the human intestine. These transporter proteins included FABPpm (panel A), FATP4 (panel B) and FAT/CD36 (panel C). Panel D displays Western Blots of the different segments of a single subject, which is representative for the average distribution pattern of FABPpm and FATP4. * = P<0.05 compared to duodenum; # = P<0.05 compared to proximal colon; $ = P<0.05 compared to distal colon. D = duodenum, J = jejunum, I = ileum, PC = proximal colon and DC = distal colon.

FABPpm protein level was significantly higher in ileum (1.04±0.13) than in proximal colon (0.64±0.07; P = 0.026) and distal colon (0.66±0.09; P = 0.037), and tended to be higher in duodenum than in proximal and distal colon (P = 0.059 and P = 0.082 respectively) (figure 4A). For FATP4, no significant differences were found between any of the segments (figure 4B). FAT/CD36 protein level was significantly higher in ileum (2.03±0.42), when compared to that in duodenum (P = 0.017), proximal colon (0.89±0.13; P = 0.011) and distal colon (0.97±0.15; P = 0.014) (figure 4C). Comparable to the sterol transporters, no significant differences were found in intestinal LCFA transporter levels between men and women and no correlations with age could be shown. Blots of individual subjects representative for the group are presented in figure 4D.

## Discussion

### Sterol transporters

Since intestinal cholesterol absorption is tightly regulated by a number of transporter proteins (ABCA1, ABCG5, ABCG8, and NPC1L1) we decided to describe for the first time distribution profiles of these transporter proteins along the duodenal-colonic axis in humans, as a first step to further understand *in vivo* human intestinal cholesterol metabolism. In contrast to humans, some animal data on the importance of different segments for intestinal cholesterol metabolism is available. For example, Sylven *et al*. [33] have reported that in rats the major part of cholesterol was taken up in the proximal half of the small intestine, when cholesterol was administered in crystallized form. When administered in an oil phase, the gross uptake occurred more distally. In humans, cholesterol is first incorporated into micelles in the duodenum. Thus, if these animal data can be extrapolated to the human situation, it suggests that cholesterol absorption in humans mainly occurs in the distal part of the small intestine. From another study in 29 obese subjects undergoing Roux-en-Y gastric bypass [34], it was concluded that cholesterol absorption decreased significantly after bypassing the proximal intestine. However, uptake of cholesterol was not completely abolished, suggesting that the more distal regions of the intestine are also capable of absorbing cholesterol. On the other hand, from *in vivo* studies with porcine intestine, it was concluded that the predominant site for cholesterol uptake was the jejunum [35], [36], [37]. Thus, results from animal and human studies are not uniform and it was therefore a priori unclear in which segment the protein level of cholesterol-transporter proteins like NPC1L1, ABCG5 and ABCG8 would be most abundant. We now found that the levels of NPC1L1 and ABCG8 were bell-shaped along the proximal-distal axis, with the highest levels in ileum, suggesting that transporter-dependent sterol fluxes in humans could be located more distally than in pigs and rodents.

For mRNA, Davies *et al*. [38], showed that NPC1L1 expression was very similar in jejunum, ileum and duodenum, but higher in these segments than in segments of the large intestine. This may suggest that protein levels do not parallel mRNA expression. Another study in rats found the highest NPC1L1mRNA and protein levels in jejunum [39]. Similarly, Sané and coworkers [17] found the highest levels of NPC1L1 protein in jejunum in human subjects. The differences between this human study and our results might be explained by the difference in subject population. The samples in the study by Sané *et al*. were partially derived from patients with Crohn's Disease, which are known to display abnormalities in lipid metabolism [40], [41], which presumably might coincide with alterations in transporter expression levels and distribution patterns. Finally, it should be realized that a lot of the data available in the literature is derived from animal studies. In this respect, species-specific differences in organ-specific transport protein mRNA levels between mouse, rat and human tissue have been shown [39]. This may be an additional reason for different transporter distribution patterns within the intestine between humans and other species.

Because of the co-localization of ABCG5 and ABCG8 towards the apical membrane [42], [43], [44], a similar distribution pattern of these two transporters was expected. However, ABCG5 levels were very similar between the intestinal segments and did not correlate with ABCG8 levels, for which we do not have an explanation.

ABCA1 plays a role in the biogenesis of anti-atherogenic high-density lipoproteins (HDL) in the intestinal lining [45], [46], [47]. Using enterocyte specific ABCA1 knock-out mice, Brunham *et al.* [48] showed that in mice approximately 30% of the steady state plasma HDL pool was due to intestinal ABCA1 activity. According to our data, ABCA1 protein levels were the highest in ileum. A similar distribution has been described for ABCA1 mRNA in hamsters [49]. This may suggest that the ABCA1-mediated assembly of HDL in the human intestine mainly occurs in ileum.

Except for levels (expressed per mg of total protein) of the active transporters, the actual absorption also depends on the surface areas of the different segments. Estimates for the duodenum are 0.1 m^2^, for jejunum 60 m^2^, for ileum 60 m^2^, and for colon 0.2 m^2^ [50], [51]. Combining these areas with the segmental transporter levels, it can now be hypothesized that the ileum is the most important site for transporter-dependent sterol fluxes. Moreover, based on the finding that bowel resection of the proximal 75% of the pig small intestine led to adaptive absorption of cholesterol in ileum [35], it is tempting to speculate that also the dietary availability of fatty acids and cholesterol is important in understanding these processes. Relatively, intraluminal availability of (dietary) cholesterol and LCFA is highest in duodenum, where a lot of fatty acids and cholesterol can easily be absorbed by the relevant transport proteins and also passive diffusion of FA is more eligible to occur. More distally, relative cholesterol and fatty acid bioavailability is lower, and therefore more transport proteins are required for the same absolute uptake of cholesterol.

### Sterol and LCFA transporters

Recent studies have suggested that the LCFA transporter FAT/CD36, like the cholesterol transporter NPC1L1 a member of the CD36 superfamily, plays a crucial role in cholesterol uptake in the proximal intestine of mice [18]. In FAT/CD36 deficient mice, *in vitro* cholesterol uptake in the first of three equal intestinal segments was approximately 75% lower as compared to that in wild-type littermates. For the second and third segment no differences were found. The distribution pattern of FAT/CD36 was comparable to the pattern found for NPC1L1, but the highest protein expressions were found more distally than would be expected based upon the hypothesis of their role in proximal cholesterol uptake, however the patterns found in this study for NPC1L1 and FAT/CD36 were comparable.

In a different study on the role of FA transporters in cholesterol transport [19], overexpression of cytoplasmic I-FABP in a human intestinal epithelial cell line not only resulted in a decrease in free cholesterol absorption from micelles, but also in a downregulation of NPC1L1, and upregulation of ABCA1, ABCG5 and G8. These findings suggest that overexpression of cytosolic I-FABP favors cholesterol efflux.

Expression of transporter molecules may be changed by the composition of the diet, as in mice the FABP protein level in the distal small intestine was increased after high-fat intakes [52]. Similar findings were shown for FAT/CD36 after consumption of LCFAs, but not after consumption of MCFAs [53]. These studies clearly indicate a role for nutrition in regulating transporter mRNA and possibly also protein levels. Unfortunately, we have no data records on dietary habits of the subjects. De Vogel-van den Bosch *et al*. [20] studied the possible interrelations between FA and cholesterol uptake *in vivo*. A cholesterol-free, high-fat diet suppressed gene expression of the cholesterol transporters NPC1L1, ABCA1, ABCG5 and ABCG8 in the middle segment of the mouse small intestine after 2, 4 and 8 weeks. Thus, these studies suggest that an interrelation exists between FA and cholesterol uptake, potentially mediated by changes in intestinal transporter activity. Although these interrelations are not completely understood, it does suggest that knowledge on distribution profiles of LCFA transporters in combination with those of sterol transporters is needed to optimize potential intervention strategies to lower sterol absorption.

Besides playing a potential role in intestinal cholesterol uptake, FAT/CD36 is also involved in a wide range of physiological processes and disorders [54]. FAT/CD36 in particular is important for very long-chain (VLC) FA (more than 18 carbon atoms) metabolism, since intestinal VLCFA uptake was completely abolished in CD36^−/−^ mice [55].

Lobo *et al.* [56] described that in humans, FAT/CD36 protein was restricted to duodenal and jejunal epithelium. On the other hand, Poirier *et al*. [53] also found FAT/CD36 protein in rat ileal epithelium, although in lower amounts, with the highest FAT/CD36 protein expression in jejunum, followed by duodenum. In both of the studies, membrane proteins were separated from the lysates and then further analyzed. In contrast, Chen *et al.* [57] used total cell lysate, and found a distribution pattern comparable with that of Poirier *et al*. [53], but also found FAT/CD36 protein in the stomach and colon of rats. Another study by Nassir *et al.* [18] found the highest levels of FAT/CD36 in rats in duodenum, with a steep decrease in expression when proceeding more distally. Because of the apparent discrepancies in transporter expression profiles between species, Wang *et al*. [58] analyzed tissues of both rats and humans. They used total lysates – in line with Chen *et al*. [57] - and showed ubiquitous CD36 mRNA and protein in all intestinal segments of the rat. In the human samples the mRNA distribution was different from FAT/CD36 protein, since FAT/CD36 protein was highest in ileum, while mRNA was lower in ileum than in duodenum, jejunum and colon. Our data closely resemble the protein data from Wang *et al*., in which the same technique for tissue preparation was used. The above data indicates that there are differences in the distribution patterns of CD36 between different species and that, at least in humans, protein levels do not resemble mRNA levels, a finding of which there are numerous other examples as reviewed by Glatz *et al*. [59]. Our results suggest a more distally situated (ileum) FAT/CD36-mediated uptake of LCFA in humans than in rats. Additionally, we found a small amount of FAT/CD36 in colon, but levels were relatively low.

Concerning other fatty acid transporters, it was suggested that FABPpm is co-expressed with FAT/CD36 [58]. Indeed, there are several indications for an interaction between both transporters at the protein level [60], [61]. As levels were the highest in the ileum, our results suggest that the small intestine is more important than the large intestine in FABPpm mediated LCFA uptake. Co-expression of FABPpm and FAT/CD36 is not suggested by our data, since we did not find significant correlations for any of the segments.

Also FATP4 is important in LCFA transport [23], [62]. In mice, Stahl *et al*. [23] showed the highest expression of FATP4 in jejunum and ileum, but there were also detectable amounts present in duodenum. These findings are partly confirmed by our study, since we found FATP4 in all segments. The presence of FATP4 in human colon is not in agreement with the findings of Stahl *et al*. [23]. We suggest that the presence of fatty acid transporters in the distal intestine is a last resort for the absorption of LCFA. In fact, fatty acid uptake is very efficient and approximately 90–95% of dietary fatty acids are absorbed. Most of the FA uptake is probably complete in the proximal small intestine [63], [64], but also ileum is capable of absorbing FA [65], [66], which is in line with the locations of the LCFA transport proteins in combination with estimations of segmental surface area.

### Gender and age differences

Duan *et al*. [67] showed that estrogens may influence cholesterol uptake through up-regulation of NPC1L1 and possibly by down-regulation of the sterol efflux transporters ABCG5 and ABCG8. It was suggested that estrogen acts as transcription modulator for the target genes through effects on estrogen receptor (ER) α or ER β, two subtypes of the steroid hormone receptor superfamily. Furthermore, a significant positive effect of aging on cholesterol uptake through inhibition of the sterol efflux transporters ABCG5 and ABCG8 has been reported [67]. In contrast, fat uptake was suggested to decline with ageing [68], [69], [70] and also more recently decreased ileal uptake of palmitic, stearic, oleic and linoleic acid was found with ageing in rats [71]. These effects were abolished when mucosal surface area was considered. Other studies however have suggested that age is not related to lipid absorption [72] or to morphological changes of the intestine [73], whilst others have even reported an increased lipid uptake with ageing [74]. We did not find a decrease in any of the lipid transport proteins with ageing, nor did we find gender-related differences. However, since the women were probably post-menopausal, no estrogen-related gender difference was expected. Concerning the influence of gender and age, the power of the study may not have been sufficient or the range of age may not have been large enough.

### Conclusion

In conclusion, this is the first study that has analyzed the distribution of cholesterol and LCFA transport proteins along the entire human duodenal-colonal axis. Protein distribution patterns are different from the sites suggested for predominant uptake of cholesterol and LCFA in pigs, mice and rats [18], [33], [35], [36]. Our expression data, combined with recent physiological *in vivo* observations in humans suggest also a prominent role for more distal parts, i.e. ileum, in transporter-mediated uptake of at least cholesterol.

This strengthens the assumed differences in species-specific expression patterns. Moreover, also the discrepancy between the distribution patterns of ABCG5 and ABCG8 was not expected. Unfortunately, we were not able to determine the subcellular distribution of cholesterol and LCFA transporters or protein activities. Altogether these findings indicate that further research in this field is warranted.

## Materials and Methods

### Ethics Statement

The study had been approved of by the respective local medical ethical committees.

### Human tissue preparation

Human intestinal tissue samples were obtained from autopsies of 7 female and 4 male subjects, aged 37 to 83 years (62.8±5.0 (mean±SEM); Medical University of Bialystok, Bialystok, Poland and Mental Hospital, Choroszcz, Poland), 18.1±1.5 h after death (table 1). The subjects died of non-intestine related diseases, such as cardiac, cerebral, renal or pulmonary events. Samples, which were directly frozen in liquid nitrogen, were taken from the duodenum (10 cm from pylorus), jejunum (50 cm from pylorus), ileum (15 cm from ileocecal junction), proximal colon (5 cm from ileocecal junction) and distal colon (10 cm from splenic flexure). Subsequent sample preparations were performed at 4°C.

**Table 1:** Subject information on age at death and biopsy time (mean±SEM).

	men	women	all	p
N	4	7	11	-
age at death	57.8±9.4	65.7±5.9	62.8±5.0	0.469
time of biopsy	20.0±2.8	17.0±1.7	18.1±1.5	0.360

Before analyses, tissues were homogenized (3–16% w/v) in SET-buffer (0.25 M sucrose, 10 mM EDTA, 10 mM Tris, pH 7.4) using an Ultra-Turrax homogenizer (IKA Werke, Breisgau, Germany) and sonificated on ice (4×15 s, MSE ultrasonic disintegrator). Total crude homogenate samples were stored at −80°C until analyses. Protein concentrations in the tissue homogenates were quantified with the Pierce micro-BCA assay (Pierce, Rockford, USA).

### Western blot analyses of human FABPpm, FATP4, ABCG5/G8, ABCA1 and NPC1L1

For detection of FABPpm, tissue samples containing 30 µg protein were loaded on SDS-PAGE precast gels (4–15% gradient gel, Tris-HCl, 1.0 mm, Biorad, Hercules, USA) applying the Criterion™ system (Biorad, Hercules, USA). For detection of ABCG5, ABCG8 and FATP4, self-cast gels were prepared with a 7.5% gradient. For ABCA1 and NPC1L1 detection, a gel with a gradient of 5% was prepared. Samples were electrophorezed (90 min, 200 V) and blotted on nitrocellulose (0.45 µm) at 4°C. After blotting, membranes were blocked overnight at 4°C with either 5% non-fat dry-milk (NFDM) in TBS-Tween 20 0.1% (TBS-T) for FABPpm, ABCG5, ABCG8 and FATP4, and 3% NFDM in TBS-T for ABCA1 and NPC1L1. After blocking, all blots were washed 5 times with TBS-T and incubated for 1 h with the primary antibodies polyclonal goat anti-FATP4 (prepared, purified and characterized by dr. J. Füllekrug, Department of Gastroenterology, University Hospital Heidelberg, Germany), polyclonal anti-ABCG5 and anti-ABCG8 (Santa Cruz Biotechnology, CA, USA), polyclonal rabbit anti-FABPpm (kind gift from dr. Calles-Escandon, Department of Medicine, University of Vermont, Burlington, Vermont), monoclonal mouse anti-ABCA1 (kind gift from dr. A.K. Groen, Department of Pediatrics/Laboratory Medicine, University Hospital Groningen, Groningen, The Netherlands), polyclonal rabbit anti-NPC1L1 (Cayman Chemical, MI, USA) in either 5% non-fat dry-milk (NFDM) in TBS-Tween 20 0.1% (TBS-T) for FABPpm, ABCG5 and G8 and FATP4, and 3% NFDM in TBS-T for ABCA1 and NPC1L1. Following overnight blocking, secondary antibodies were added and incubated for 60 min. Detection of ABCG5/G8, FABPpm, FATP4, ABCA1 and NPC1L1 was performed by enhanced chemi-luminescence (ECL). Blots were subsequently analyzed by Quantity One software (Biorad, Hercules, USA).

### FAT/CD36 ELISA

FAT/CD36 protein level in the different segments was measured via a sandwich ELISA as described previously [75]. In short, a polyvinylchloride microtitre plate (Falcon type 3912, Beckton Dickinson, Oxnard, CA) was coated overnight and washed five times. Thereafter, wells were blocked for 30 min with PBS/2% Marvel. Following 5 washing steps with PBT, standard containing 0–1 µg/ml recombinant 6-His FAT in PBS/0.4%Triton X-100 was added per well. The samples were diluted 1∶1 and added to the plate after centrifugation. After incubation for 90 min, wells were washed. Then, phage (2×10^11^ colony forming units (cfu)/well in PBS/2% Marvel) were added and incubated for 90 min. After 5 washes, 1/5000 diluted sheep anti-fd labeled with horseradish peroxidase in PBS/2% Marvel was added. After one-hour incubation and 5 washes, plates were developed with 100 µl TMB/well. The reaction was stopped after 10 min and the absorbance was read at 450 nm using a Titertek Multiscan MkII microplate reader. The CD36 ELISA showed a detection limit of 50 µg/L. Using standards of 2000 and 1000 µg/L, the intra-assay and inter-assay coefficients of variation (CV) were <10% and 15%, respectively.

### Standardization

Protein level of each lane was standardized by loading 30 µg of protein for every sample. A duodenal biopsy sample was included in every blot as an internal standard and levels of intestinal protein level of all biopsy samples loaded on the blots were corrected for the variations in the internal standards. Furthermore, duodenal transporter level was set at 1 and levels in the other segments were compared to that in duodenum.

Since intestinal samples contained both enterocytes and myocytes, we also measured H-FABP as a marker of muscle content with a direct non-competitive sandwich-type ELISA using monoclonal antibodies obtained from Hycult biotechnology (HK 403; Uden, the Netherlands) as described previously [76]. These data showed significantly higher H-FABP in jejunum (1.73±0.30) as compared to duodenum, ileum (1.09±0.19), proximal colon (0.97±0.16) and distal colon (0.90±0.17), Because of this, we concluded that the biopsy muscle content was for an unknown reason higher in our jejunum biopsies and this might have resulted in an underestimation of cholesterol transport protein levels, which are only expressed in enterocytes. Therefore cholesterol transport protein levels were corrected for the muscle content as measured via hFABP levels. Because FA transport proteins are also present in myocytes, differences in distribution patterns of the fatty acid transporters could have been influenced by the higher levels of myocytes in our jejunal biopsies. However, it is not possible to correct FA transporter levels for this possible confounding effect, because of their presence in myocytes and we do not know whether this correction might lead to overestimation or underestimation of the transport protein levels.

### Statistical analysis

Data are presented as mean ± SEM. All protein levels were related to the duodenal protein level. All data were normally distributed as tested with Shapiro-Wilk's test for normality. Analysis of variance (ANOVA) was used to compare mean transporter levels between the various segments of the intestine. Alpha inflation due to multiple comparisons was corrected with Tukey's HSD Post Hoc tests and P<0.05 was considered to be statistically significant. Independent sample t-tests were used to compare transporter levels between men and women within each segment. Pearson's correlations were determined to evaluate the possible relationship between ageing and intestinal transport protein level within each segment. Moreover, linear regression analysis was performed regarding time of biopsy after death versus expression levels of ABCA1, ABCG5, ABCG8, NPC1L1 and FAT/CD36 to evaluate whether protein degradation was an issue.

All tests were performed with SPSS 16.0 (SPSS Inc. Chicago, Illinois).
